# Trimethylamine N-oxide: a meta-organismal axis linking the gut and fibrosis

**DOI:** 10.1186/s10020-024-00895-8

**Published:** 2024-08-23

**Authors:** Jae Woong Jang, Emma Capaldi, Tracy Smith, Priyanka Verma, John Varga, Karen J. Ho

**Affiliations:** 1 Department of Surgery, Feinberg School of Medicine, Northwestern University, 676 North St. Clair Street, Suite 650, Chicago, IL 60611 USA; 2 Department of Internal Medicine, University of Michigan, 1500 East Medical Center Drive, Floor 3, Reception A, Ann Arbor, MI 48109 USA

**Keywords:** Trimethylamine, Trimethylamine N-oxide, Gastrointestinal microbiome, Choline, Carnitine, Renal insufficiency, chronic, Fibrosis, Metabolic dysfunction-associated steatotic liver disease, Metabolic dysfunction-associated steatohepatitis, Heart failure

## Abstract

**Background:**

Tissue fibrosis is a common pathway to failure in many organ systems and is the cellular and molecular driver of myriad chronic diseases that are incompletely understood and lack effective treatment. Recent studies suggest that gut microbe-dependent metabolites might be involved in the initiation and progression of fibrosis in multiple organ systems.

**Main body of the manuscript:**

In a meta-organismal pathway that begins in the gut, gut microbiota convert dietary precursors such as choline, phosphatidylcholine, and L-carnitine into trimethylamine (TMA), which is absorbed and subsequently converted to trimethylamine N-oxide (TMAO) via the host enzyme flavin-containing monooxygenase 3 (FMO3) in the liver. Chronic exposure to elevated TMAO appears to be associated with vascular injury and enhanced fibrosis propensity in diverse conditions, including chronic kidney disease, heart failure, metabolic dysfunction-associated steatotic liver disease, and systemic sclerosis.

**Conclusion:**

Despite the high prevalence of fibrosis, little is known to date about the role of gut dysbiosis and of microbe-dependent metabolites in its pathogenesis. This review summarizes recent important advances in the understanding of the complex metabolism and functional role of TMAO in pathologic fibrosis and highlights unanswered questions.

## Background

Fibrosis is a pathologic process characterized by excessive deposition of extracellular matrix (ECM; all abbreviations are shown in Table 1) in response to injury, which leads to organ dysfunction and failure. However, formation of fibrotic tissue is also fundamental for tissue response to injury. After injury, fibroblasts become activated, increase their contractility, secrete inflammatory mediators, and synthesize ECM such as collagen or fibronectin, which then results in normal wound healing and repair. When there is severe injury, chronic inflammation, or dysregulation of the wound healing response, ECM deposition becomes excessive, leading to abnormal tissue architecture and organ dysfunction or failure. Fibrosis can affect any organ system and is ultimately responsible for up to 45% of all deaths in industrialized nations (Henderson et al. 2020).

**Table 1:** Abbreviations are presented in alphabetical order

Akt	protein kinase B
ATF6	activating transcription factor 6
ATP1B1	ATPase Na+/K + transporting subunit beta 1
CKD	chronic kidney disease
*CntA*	carnitine monoxygenase
*CutC*	choline utilization gene choline-TMA lyase
CYP7A1	cholesterol 7 alpha hydroxylase
DMB	3,3-dimethyl-1-butanol
ECM	extracellular matrix
FMO3	flavin-containing monooxygenase 3
GC-C	receptor guanylyl cyclase C
*GrdH*	betaine reductase complex component B subunit beta
HFpEF	heart failure with preserved ejection fraction
HFrEF	heart failure with reduced ejection fraction
IL	interleukin
IMC	iodomethylcholine
IRE1	inositol-requiring enzyme type 1
MASLD	metabolic dysfunction-associated steatotic liver disease
MASH	metabolic dysfunction-associated steatohepatitis
mTOR	mammalian target of rapamycin
miRNA	microRNA
NLRP3	nucleotide-binding domain, leucine-rich-containing family, pyrin domain-containing-3
NT-proBNP	N-terminal pro b-type natriuretic peptide
PERK	protein kinase R-like endoplasmic reticulum kinase
siRNA	silencing RNA
SSc	systemic sclerosis
TGF-ß	transforming growth factor-beta
TMA	trimethylamine
TMAO	trimethylamine N-oxide
UPR	unfolded protein response

The fibrogenic response involves many cell types and molecular pathways. Myofibroblasts and fibroblasts, responsible for homeostasis of the ECM (LeBleu et al. 2013; Caam et al. 2018), are considered key effectors in fibrosis (LeBleu et al. 2013; Hinz et al. 2012). Myofibroblasts possess microfilaments that consist of alpha-smooth muscle actin, which allows them to contract (Caam et al. 2018) and transmit contractile forces to the surrounding ECM through specialized focal adhesions containing transmembrane integrins (Duscher et al. 2014). Mechanical forces promote a pro-fibrotic environment via fibroblast secretion of inflammatory mediators and recruitment of inflammatory cells. (Wong et al. 2011) Pathological myofibroblasts originate from a variety of lineages local resident fibroblasts, including bone marrow-derived inflammatory cells, circulating and/or resident mesenchymal stromal stem cells, preadipocytes, vascular mural cells (pericytes) and endothelial or epithelial cells (through endothelial or epithelial-mesenchymal transition). (LeBleu et al. 2013; Rosenbloom et al. 2017)

There is accumulating evidence that microbiota—including those resident in the oral cavity, (Bai et al. 2022) lung, (O’Dwyer et al. 2019) and gut (Xu et al. 2023)—exert remote effects on the molecular pathways governing tissue fibrosis throughout the body. However, the exact mechanisms of these interactions are not yet known. Gut microbiota produce trimethylamine (TMA), which is subsequently converted in the host liver and other tissues to trimethylamine N-oxide (TMAO), a multifunctional molecule that has profibrotic activity. This review summarizes the current state of knowledge of the role and mechanism of the meta-organismal microbe-TMA-TMAO axis in fibrosis and highlights potential therapeutic targets in the prevention or control of pathological fibrosis.

## TMAO metabolism

**Fig. 1:**
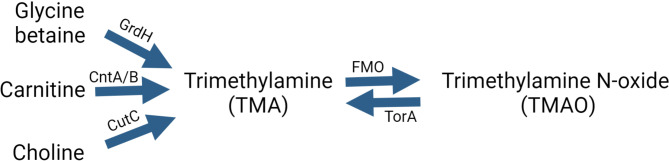
Pathways of trimethylamine (TMA) formation by gut microbiota and cycling of TMA to trimethylamine N-oxide (TMAO). GrdH, glycine betaine reductase. CntA/B, carnitine monooxygenase. CutC, choline-TMA lyase. FMO, flavin-containing monooxygenase. TorA, trimethylamine N-oxide reductase

Animal-based diets are abundant in nutrients such as choline, phosphatidylcholine, betaine, and L-carnitine, which undergo conversion by gut microbes into TMA. (Tang et al. 2015a, b) In mammals, the gut bacterial genes encoding enzymes that catalyze TMA production include choline-TMA lyase (*CutC*), carnitine monooxygenase (*CntA/B*), and glycine betaine reductase (*GrdH*) (Rath et al. 2019) (Fig. 1). The TMAO reductase pathway, which is responsible for the retroconversion of TMAO to TMA, is catalyzed by *TorA* (Mejean et al. 1994) (Fig. 1). The TMAO reductase pathway is the most prevalent in the human gut, with Proteobacteria (mainly *Klebsiella* and *Escherichia*) contributing most of the TMAO reductase sequences, (Jameson et al. 2016) while *CutC* and *GrdH* are associated with Firmicutes. In a metagenomic analysis of 50 human fecal samples, *CutC* amplicons were found in all individuals but only 26% had *CntA.* (Rath et al. 2019) Germ-free and antibiotic-treated animal models have been shown to lack the capacity for TMAO production, thus providing evidence for the role of the microbiome in the metabolism of carnitine to TMA and TMAO. (Tang and Hazen 2017; Yap et al. 2008)

Once TMA enters the host portal circulation, it is converted to TMAO, with flavin-containing monooxygenase 3 (FMO3) responsible for metabolizing the rate-limiting step. (Krueger and Williams 2005) FMO3 is predominantly expressed in the liver. However, it is also expressed in non-hepatic tissues including the lung, kidney, and brain, and we recently found, the skin. (Dolphin et al. 1996; Zhang and Cashman 2006) Systemic levels of TMA are low. (Al-Waiz et al. 1987) In humans, the capacity for TMA and TMAO production is increased in carnivores and omnivores compared to vegetarians, which is attributable to reduced or absent levels of *CntA/B* and *CutC*. (Rath et al. 2019) Approximately 50% of consumed TMAO passes through the body and is excreted in the urine. TMAO reductase can convert the remaining dietary TMAO into TMA. (Kwan and Barrett 1983) TMAO can also be consumed from dietary sources and be retroconverted to TMA. (Loo et al. 2022) Thus, systemic levels of TMAO are governed by several factors, including the diet, (Boutagy et al. 2015; Wang et al. 2019) composition and function of gut microbiota, (Rath et al. 2017) medication use, (Milks et al. 2018) FMO3 expression and activity, (Bennett et al. 2013) and renal excretion. (Janeiro et al. 2018) Due to redundant and overlapping regulatory pathways and exposure variables, plasma TMAO has high inter- and intra-individual variability. (Papandreou et al. 2020)

## Fibrotic conditions and TMAO

**Fig. 2:**
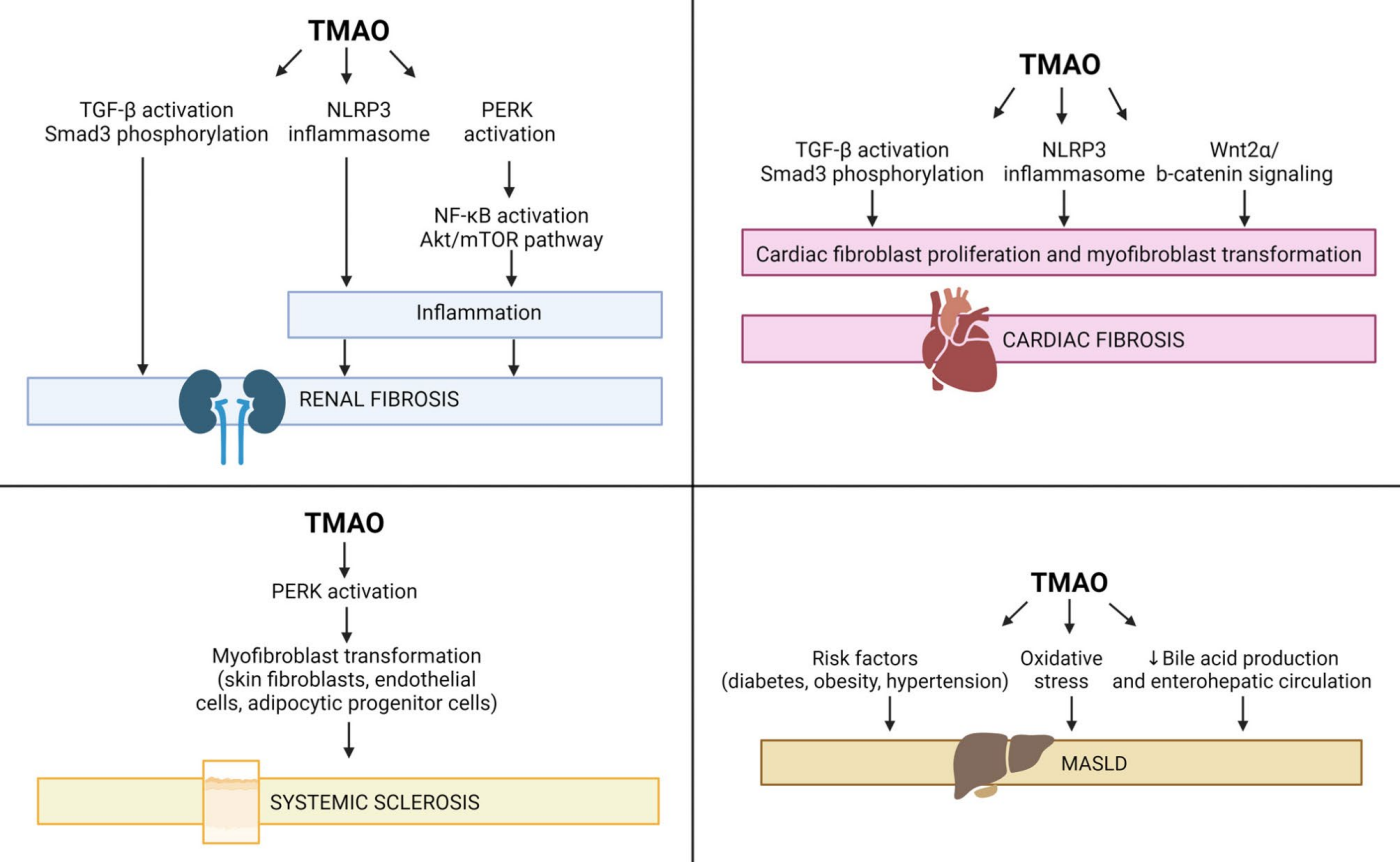
Overview of proposed mechanisms of TMAO-mediated fibrosis in the kidney, heart, liver, and systemic sclerosis. Details of mechanisms are provided in the text

A wide spectrum of human diseases is characterized by chronic inflammation leading to organ or tissue fibrosis. (Rosenbloom et al. 2017) As summarized below, TMAO has been implicated in kidney, heart, liver, and skin fibrosis. Organ-specific pathways for the pro-fibrotic effects of TMAO are shown in Fig. 2.

### Kidney

High levels of circulating TMAO are associated with concurrent adverse cardiovascular disease events including heart attack, stroke, and mortality in people with chronic kidney disease (CKD). (Kim et al. 2016; Missailidis et al. 2016; Tang et al. 2015a, b) In one prospective cohort study of 521 patients with CKD, TMAO was associated with a 2.8-fold dose-dependent increase in 5-year all-cause mortality. (Tang et al. 2015a, b) TMAO is also associated with the development of CKD. In a prospective cohort study of 1,434 people with normal renal function, patients who later developed CKD were found to have a 33% higher level of plasma TMAO compared to those who did not. (Rhee et al. 2013) In mouse and rat studies, dietary supplementation with choline or TMAO led to elevated levels of plasma TMAO, which is associated with both spontaneous (Tang et al. 2015a, b) and induced (Fang et al. 2021) kidney fibrosis and renal functional impairment.

Numerous animal studies have investigated inhibition of gut microbial TMA generation as an intervention to preserve renal function. In mice, oral administration of iodomethylcholine (IMC), a novel choline TMA-lyase inhibitor, markedly suppressed plasma TMAO levels, significantly reduced the decline in renal function, and reduced renal fibrosis in an isoproterenol model of CKD. (Gupta et al. 2020) In a separate study, investigators used methylcholine, an inhibitor of TMA lyase that functions similarly to IMC, and found attenuation of adenine-induced markers of CKD, including renal cortical scarring and collagen deposition following methylcholine treatment. Additionally, methylcholine supplementation significantly decreased expression of renal inflammatory and pro-fibrotic genes induced by adenine supplementation. (Zhang et al. 2021) Other studies have investigated inhibition of TMA production using 3,3-dimethyl-1-butanol (DMB), another TMA lyase inhibitor. (Sun et al. 2017; Zou et al. 2021) Oral DMB reduced plasma TMAO levels and prevented both renal and cardiac injury in rats with cardiorenal syndrome following induction of myocardial infarction and kidney damage. TMAO-associated increases in expression of pro-fibrotic markers TGF-ß1 and collagen I and pro-inflammatory cytokines interleukin (IL)-6 and IL-8 were attenuated by DMB. (Zou et al. 2021)

There are multiple potential mechanisms for the profibrotic role of TMAO in the kidney. SMAD3 is a member of the SMAD family of transcriptional regulators downstream of TGF-ß signaling, (Zimmerman and Padgett 2000; Kretzschmar and Massague 1998) a pathway that regulates proliferation, differentiation, and apoptosis, and tissue fibrosis. (Massague 2012; Bottinger 2007) SMAD3 phosphorylation is an important regulatory step in renal fibrosis in CKD. (Qu et al. 2014) In mice fed diets supplemented with choline and TMAO, there was increased phosphorylation of SMAD3 in the kidney. (Tang et al. 2015a, b) Mice with high fat diet-induced obesity had elevated plasma TMAO levels, increased renal fibrosis, and increased SMAD3 phosphorylation, all of which were reversed with DMB treatment. (Sun et al. 2017)

The nucleotide-binding domain, leucine-rich-containing family, pyrin domain-containing-3 (NLRP3) inflammasome is another pathway that may mediate TMAO-induced renal fibrosis. In rat models of diabetic kidney disease, dietary supplementation with TMAO and choline were found to increase activation of NLRP3 inflammasome, leading to increased IL-1β and IL-18 secretion, renal inflammation, oxidative stress, and reactive oxygen species (ROS) formation. (Fang et al. 2021) In human renal fibroblasts, TMAO stimulation led to increased cell proliferation and NLRP-3 and caspase-1 protein expression in these cells. Knocking out NLRP-3 and caspase-1 prevented TMAO-dependent cell proliferation. Furthermore, inhibitors of protein kinase R-like endoplasmic reticulum kinase (PERK), protein kinase B (Akt), and mammalian target of rapamycin (mTOR) each attenuated renal fibroblast proliferation and collagen production, suggesting the mechanistic role for each in mediating the effect of TMAO on renal fibroblast proliferation. (Kapetanaki et al. 2021) Finally, TMAO enhanced TNF-α mediated renal inflammation by inducing the release of cytokines, chemokines, inflammatory mediators, and growth mediators from renal fibroblasts. (Stefania et al. 2024) Based on these findings and prior studies that have demonstrated the association between NLRP3 inflammasome, Akt, mTOR, and TMAO, (Artlett 2012; Artlett and Thacker 2015; Boini et al. 2017; Zhao et al. 2020; Gallego et al. 2020; Li et al. 2018a, b) the mechanisms underlying the effect of TMAO on renal fibrosis include TMAO binding to and activating PERK, which triggers the Akt/mTOR pathway, which, in turn, promotes cell proliferation and collagen production or regulates NLRP3 and caspase-1 to indirectly promote cell proliferation. Activated PERK may also directly regulate NLRP3 through NF-κB activation. (Kapetanaki et al. 2021)

Finally, progression of CKD and augmentation of kidney fibrosis by TMAO may involve two additional mechanisms: (1) upregulation of SMAD3/transforming growth factor-beta (TGF-ß) signaling and (2) generation of reactive oxygen species (ROS). The relationship between TGF-ß signaling and ROS has been well-documented in cancer biology, with studies suggesting that ROS activates latent TGF-ß to its active form. (Chung et al. 2021) Whether similar links are implicated in renal fibrosis is an area of intriguing future research.

### Heart

Myocardial fibrosis, or the deposition of excess ECM in the cardiac interstitium, can be both reparative and pathologic. For example, after myocardial infarction, reparative fibrosis preserves the structural integrity of the heart. (Frangogiannis and Kovacic 2020) However, interstitial fibrosis secondary to systemic hypertension can lead to increased myocardial stiffness and diastolic dysfunction. (Diez et al. 2020) Gut microbial metabolites, including TMAO, (Guo et al. 2020; Troseid et al. 2015; Krack et al. 2005; Anker et al. 1997; Dong et al. 2021) and their precursors such as betaine and choline (Papandreou et al. 2021) have been associated with increased cardiovascular disease including heart failure (Guo et al. 2020; Troseid et al. 2015; Krack et al. 2005; Anker et al. 1997; Dong et al. 2021) and atrial fibrillation. (Papandreou et al. 2021)

Elevated systemic TMAO is associated with increased risk of major adverse cardiovascular events and mortality. (Heianza et al. 2017; Tang et al. 2013) This association persists in patients with heart failure. One meta-analysis involving nearly 7,000 patients with heart failure reported an increase in all-cause death, recurrent myocardial infarctions, and rehospitalizations with increasing systemic TMAO independent of renal function, (Li et al. 2020) and this has been corroborated in multiple other large studies. (Stubbs et al. 2019; Zhou et al. 2020; Li et al. 2022)

Numerous prospective cohort studies have also found elevated plasma TMAO levels to be a predictor of cardiovascular death in patients with acute (Schuett et al. 2017; Israr et al. 2021; Kinugasa et al. 2021; Suzuki et al. 2016) and chronic heart failure, (Zhou et al. 2020; Wei et al. 2022) peripheral artery disease, (Roncal et al. 2019) and patients with end-stage renal disease on hemodialysis. (Zhang et al. 2020) Of these studies, one prospective cohort study involving 2,490 patients with heart failure with reduced ejection fraction (HFrEF) or heart failure with preserved ejection fraction (HFpEF) found plasma TMAO levels to have a better predictive value than N-terminal pro-brain natriuretic peptide (NT-proBNP), an established risk marker in heart failure without direct connections to TMAO, for mortality and cardiovascular mortality in HFrEF patients. (Schuett et al. 2017) Another prospective cohort study with 196 participants found plasma TMAO levels to be helpful for risk stratification of patients with HFpEF, especially when used in conjunction with NT-proBNP. (Salzano et al. 2020) A study of patients with coronary artery disease found an association between plasma TMAO levels, NT-proBNP, and prognosis, risk stratification, and length of hospitalization. (Qiu et al. 2022) Of note, a prospective cohort study involving Polish patients with cardiovascular disease found a statistically significant but clinically non-significant association between circulating TMAO and 5-year mortality. Furthermore, a recent large study of patients with type 2 diabetes mellitus found no significant associations between TMAO, choline, or TMA with heart failure requiring hospitalization, cardiovascular death, or all-cause death. (Wargny et al. 2022) Thus, while most studies demonstrate the clinical utility of using TMAO to establish cardiovascular disease prognosis and outcome, these latter studies suggest the need for further research to elucidate the precise role of TMAO on the mortality of patients with cardiovascular disease. (Konieczny et al. 2022)

An association between cardiovascular complications, including cardiac fibrosis, and elevated TMAO in animal models has been demonstrated by multiple investigators. (Zou et al. 2021; Li et al. 2017, 2018a, b, 2019; Organ et al. 2016; Zhang et al. 2017; Yang et al. 2019a, b; Wang et al. 2020; Strilakou et al. 2016; Shuai et al. 2020; Nanto-Hara et al. 2020; Chen et al. 2017) One study in mice found that transverse aortic constriction-induced heart failure was significantly worse in mice fed diets supplemented with choline or TMAO. (Organ et al. 2016) In other studies, mice on diets supplemented with TMAO or choline exhibited significantly more surgical and doxorubicin-induced cardiac fibrosis than those on the control diet, (Yang et al. 2019a, b; Li et al. 2019) and inhibition of TMA lyase using DMB prevented choline-related cardiac fibrosis. (Yang et al. 2019a, b) High choline diets also exacerbated myocardial fibrosis and cardiac dysfunction in a mouse model of heart failure with HFpEF. (Shuai et al. 2020) Finally, in a mouse study on the cardioprotective effects of voluntary exercise on myocardial inflammation and fibrosis, the benefits of exercise were abrogated by TMAO supplementation. (Zhang et al. 2017)

Targeting circulating TMAO levels to decrease cardiac fibrosis is an active area of investigation with mixed results to date. Treatment with DMB prevented susceptibility to ventricular arrhythmia and adverse cardiac structural remodeling in mice with overload-induced heart failure from aortic banding surgery (Wang et al. 2020) and ameliorated cardiac diastolic dysfunction, myocardial fibrosis and inflammation in a mouse model of uninephrectomy and aldosterone-induced HFpEF, (Wang et al. 2020; Shuai et al. 2020) but these mice were not given a high choline or TMAO diet. On the other hand, in mice with Western diet-induced obesity, DMB reduced plasma TMAO levels but did not alter other parameters such as cardiac inflammation, fibrosis, and dysfunction. (Chen et al. 2017) One study explored the prospect of altering microbe-produced TMAO using a guanylate cyclase C (GC-C) agonist, linaclotide, and found that linaclotide reduced TMAO and ameliorated both renal and cardiac fibrosis in a mouse model of adenine-induced CKD. (Nanto-Hara et al. 2020) As receptor guanylyl cyclase C (GC-C) signaling in the gut epithelium has been implicated as an important factor in host defense against pathogenic bacteria, (Amarachintha et al. 2018) the study proposed that the increase in GC-C signaling from linaclotide reduced the bacterial species that generate TMA. In rats subjected to myocardial infarction, Luhong granules—a multi-medicinal herb combination—reduced both TMAO and lipopolysaccharide levels through gut microbial modification and reduced intestinal pathology, which ultimately decreased adverse ventricular remodeling. (Yang et al. 2019a, b)

While numerous studies have supported the positive association between cardiovascular disease or cardiac fibrosis with elevated TMAO levels, there are other studies, both in preclinical models and in human populations, that have not found such as association. In a study using pressure-overloaded hearts in hypertensive rats, it was shown that chronic treatment with low dose TMAO reduced cardiac fibrosis. (Huc et al. 2018) Another study found that a choline-deficient diet promoted fibrosis in rats (Strilakou et al. 2016). In a rat model of spontaneously hypertensive heart failure, higher TMAO levels reduced mortality and were associated with diuretic, natriuretic, and hypotensive effects. (Gawrys-Kopczynska et al. 2020) In fact, some studies have found that low levels of TMAO may have positive effects on the heart. (Huc et al. 2018; Gawrys-Kopczynska et al. 2020; Strilakou et al. 2013) For example, one study that explored the effects of dietary TMAO supplementation in spontaneously hypertensive rats found that a 4-5-fold increase in plasma TMAO levels was associated with reduced plasma levels of NT-proBNP and vasopressin and lower left ventricular end-diastolic pressure and cardiac fibrosis—all indications of improved diastolic dysfunction. (Huc et al. 2018) Collectively, these results suggest a context-dependent causative role of TMAO in cardiac fibrosis.

The known profibrotic mechanisms of TMAO in the myocardium appear to be similar to the mechanisms in the kidney and involve TGF-β and or the NLRP3 inflammasome. TMAO treatment of primary mouse cardiac fibroblasts was found to induce a dose-dependent increase in proliferation, migration, collagen secretion, and expression of profibrotic factors, TGF-β and phosphorylated SMAD3. (Li et al. 2019) Mouse cardiac fibroblasts treated with TMAO also exhibited increased NLRP3 inflammasome activation, while silencing RNA (siRNA)-mediated knockdown of NLRP3 in cardiac fibroblasts blunted TMAO-induced cell proliferation as well as TGF-β and collagen expression. (Li et al. 2019) TMAO has also been shown to transform atrial fibroblasts into myofibroblasts through the activation of the Wnt2a/β-catenin signaling pathway. (Yang et al. 2019a, b)

### Systemic sclerosis

Systemic sclerosis (SSc) is a complex chronic autoimmune disease characterized by inflammation, vascular injury and fibrosis that synchronously affect virtually every organ system. (Allanore et al. 2015; Denton and Khanna 2017) While the precise etiology of SSc remains obscure, both genetic and environmental factors contribute. (Allanore et al. 2015) Several lines of evidence implicate gut dysbiosis in the pathogenesis of fibrosis characteristic of SSc. (Volkmann 2017; Patrone et al. 2017; Johnson et al. 2019) Multiple bacterial taxa that are enriched in the SSc gut microbiome (e.g. *Ruminococcus*) (Volkmann 2017) are known to be high TMA producers. (Wu et al. 2018) Expression of FMO3 is significantly up-regulated in both explanted skin fibroblasts, and in skin biopsies, from patients with SSc (Chadli et al. 2019; Kim et al. 2022; Skaug et al. 2020) Moreover, in a recent study, SSc patients with interstitial lung disease (ILD) and esophageal dysmotility had higher plasma TMAO levels than non-SSc controls. (Stec et al. 2023)

The endoplasmic reticulum stress kinase PERK (protein kinase R [PRK]-like endoplasmic reticulum kinase, EIF2AK3) has been identified as a potential receptor for TMAO. (Chen et al. 2019) Microarray analysis on rat hepatocytes treated in the presence or absence of pathophysiologic levels of TMAO showed that TMAO selectively induced the PERK pathway of the unfolded protein response (UPR) without upregulation of the inositol-requiring enzyme type 1 (IRE1) or activating transcription factor 6 (ATF6) pathways. In vivo studies in mice demonstrated that dietary supplementation of TMAO increased phosphorylation of PERK and increased expression of the transcription factor *Foxo1*, a key driver of metabolic syndrome, readouts which were blocked by co-administration of the PERK inhibitor GSK2656157 or liver-specific knock-out of PERK. Furthermore, direct interaction between TMAO and PERK was confirmed by isotope labeled TMAO binding to immunoprecipitated PERK. The study further identified the luminal domain of PERK as a specific domain involved in the TMAO-PERK interaction. (Chen et al. 2019) Other investigators found that TMAO leads to a dose-dependent increase in PERK activation in human fibroblasts in vitro and increased PERK phosphorylation and *Foxo1* expression in human microvascular endothelial cells. The profibrotic effects of TMAO were blocked by PERK inhibition using the PERK inhibitor GSK2606414 and by siRNA silencing. (Kim et al. 2022) However, PERK binding and activation by TMAO may be cell-specific, as PERK activation was not detected in TMAO-treated kidney epithelial cells, (Zhang et al. 2021) suggesting that further investigation of the cell-specific mechanisms of TMAO activity is warranted.

When exposed to TMAO in vitro, skin fibroblasts, vascular endothelial cells, and adipocytic progenitor cells are reprogrammed into myofibroblasts, a transition mediated via PERK. (Kim et al. 2022) Another study showed that TMAO generated from the aorta and liver phosphorylates 12 cytosolic kinases via PERK, leading to endoplasmic reticulum stress, mitochondrial stress and metabolic reprogramming to establish trained immunity in aortic endothelial cells. (Saaoud et al. 2023) These studies suggest that exposure of fibroblasts, endothelial cells and other mesenchymal cells to high levels of TMAO may be sufficient to elicit a fibrotic response, including endothelial-mesenchymal transition. However, more studies are warranted in order to fully establish the pathogenic role, and mechanism of action, of TMAO in the vascular and fibrotic pathology characteristic of SSc.

### Liver

Metabolic dysfunction-associated steatotic liver disease (MASLD), the pathologic accumulation of hepatocellular lipid, is a steadily growing global health issue that parallels the rise in patients with obesity and metabolic syndrome. (Wong et al. 2023) A subset of patients with MASLD develop metabolic dysfunction-associated steatohepatitis (MASH), characterized by varying degrees of inflammation and fibrosis in the liver. MASH can further predispose a patient to cirrhosis or hepatocellular carcinoma. (Teng et al. 2023; Huang et al. 2022)

MASLD pathogenesis is multifactorial (i.e., “multi-hit hypothesis”) and includes genetic and epigenetic factors, hormonal factors, involvement of adipose tissue, nutritional factors, and dysbiosis. (Teng et al. 2023; Huang et al. 2022; Buzzetti et al. 2016) Choline and choline/methionine-deficient diets are established means of inducing MASLD in animal models, (Buzzetti et al. 2016) with proposed mechanisms involving (1) endoplasmic reticulum stress, (2) triglyceride aggregation and deposition, (3) NLRP3 inflammasome activation, and (4) gut dysbiosis. (Vallianou et al. 2024) While MASLD induction by a choline-deficient diet in animal models might imply that low TMAO induces MASLD, studies across humans and cellular and animal models suggest a more complex relationship.

Numerous case control and cohort studies have shown a positive association between high TMAO levels and MASLD (Chen et al. 2016a, b; Shi et al. 2022; Zhao et al. 2019) and primary liver cancer. (Liu et al. 2018) In a case-control and cross-sectional analysis of 100 patients with or without MASLD, high circulating level of TMAO was found to be associated with greater severity of MASLD. (Chen et al. 2016a, b) In a prospective study of over 100 women with or without morbid obesity and with or without MASLD, circulating TMAO level was found to be significantly elevated in patients with MASLD. (Aragones et al. 2020) More nuanced findings have been observed in patients with more advanced liver pathologies, including cirrhosis. In a study of approximately 120 patients with cirrhosis and 5,000 healthy people in the PREVEND cohort study, severity of cirrhosis was found to be associated with high levels of betaine, a precursor of TMAO. Interestingly, in the patients with cirrhosis, TMAO levels increased after liver transplantation and were higher than pre-transplantation levels. (Berg et al. 2023) In a separate study of patients with cirrhosis, elevated circulating TMAO was a marker for hepatic encephalopathy. (Jimenez et al. 2010) Finally, in a case-control study of patients with MASLD and healthy controls, serum TMAO levels correlated with serum total bile acids and hepatic mRNA expression of cholesterol 7 alpha hydroxylase (CYP7A1). (Tan et al. 2019) The same study reported that mice fed a high-fat diet and treated with TMAO demonstrated impaired liver function, hepatic triglyceride accumulation and lipogenesis, and altered hepatic bile acid composition toward farnesoid X receptor-antagonistic bile acid species, while knockdown of CYP7A1 in hepatocytes blocked the effects of TMAO-induced lipogenesis, suggesting that TMAO aggravates liver steatosis by suppressing bile acid-mediated hepatic farnesoid X receptor signaling. (Tan et al. 2019)

The mechanisms by which TMAO causes MASLD in humans are varied. TMAO is related to risk factors such as diabetes mellitus and obesity. (Dehghan et al. 2020; Zhuang et al. 2019) In addition, in vitro, high fat and TMA exposure stimulates FMO gene expression and TMAO. (Shi et al. 2022) Furthermore, TMAO reduces bile acid production by suppressing CYP7A1 and CYP27A116 and restricts bile acid enterohepatic circulation by repressing the organic anion transporter and the expression of the multidrug resistance protein family. (Koeth et al. 2013; Makishima et al. 1999; Ding et al. 2018)

Animal studies suggest that the relationship between TMAO and liver fibrosis is likely complex and non-linear. Rats exposed to chronically high doses TMAO were found to have increased oxidative stress and inflammation in the liver, but there were no structural changes or evidence of increased fibrosis. (Florea et al. 2024) However, in mouse models of MASH and acute liver fibrosis, dietary TMAO supplementation was associated with decreased liver fibrosis, improved liver function, and greater gut microbiome diversity. (Zhou et al. 2022) Furthermore, ATP1B1, an astrocyte-specific isoform of the Na+/+-ATPase transmembrane pump, is upregulated in the liver endothelial cells of mice with liver fibrosis. Endothelial-specific knockout of ATP1B1 resulted in decreased expression of profibrotic factors such as C-X-C motif chemokine ligands 10 and 1 and connective tissue growth factor, while administration of a selective ATP1B1 inhibitor decreased liver fibrosis. In fibrotic mouse livers, treatment with TMAO reduced ATP1B1 protein expression. (Zhou et al. 2022) These findings suggest that TMAO may mitigate liver fibrosis through attenuation of ATP1B1 expression and consequent improvement of endothelial integrity, a potential mechanism that highlights the organ-specific relationship between TMAO and fibrosis. (Zhou et al. 2022)

In vitro data suggest alternative mechanisms for TMAO and liver fibrosis. TMAO induces release of hepatocyte-derived exosomes from mouse-derived hepatocytes, leading to endothelial uptake and downstream inflammation, apoptosis, and endothelial dysfunction via NF-κB pathway activation. (Liu et al. 2022) In a fatty liver cell model, TMAO induced gene expression of microRNAs miRNA-34a and miRNA-122, both of which are biomarkers of MASLD in humans. (Bahramirad et al. 2024) In a separate study, incubation of cells in the fatty liver cell model with TMAO led to increased lipid deposition and elevation in expression of liver fibrosis-related genes including keratin17. (Nian et al. 2023) Knockdown of keratin17 with siRNA resulted in attenuation of the observed lipid deposition and liver fibrosis. (Nian et al. 2023)

These data demonstrate a complex role for TMAO in both the development and mitigation of liver fibrosis. The variable effects of TMAO may be due cell type- and stage-specific. Further studies are needed to uncover the precise pathophysiologic relationship between TMAO and liver fibrotic diseases.

### Therapeutic strategies

**Fig. 3:**
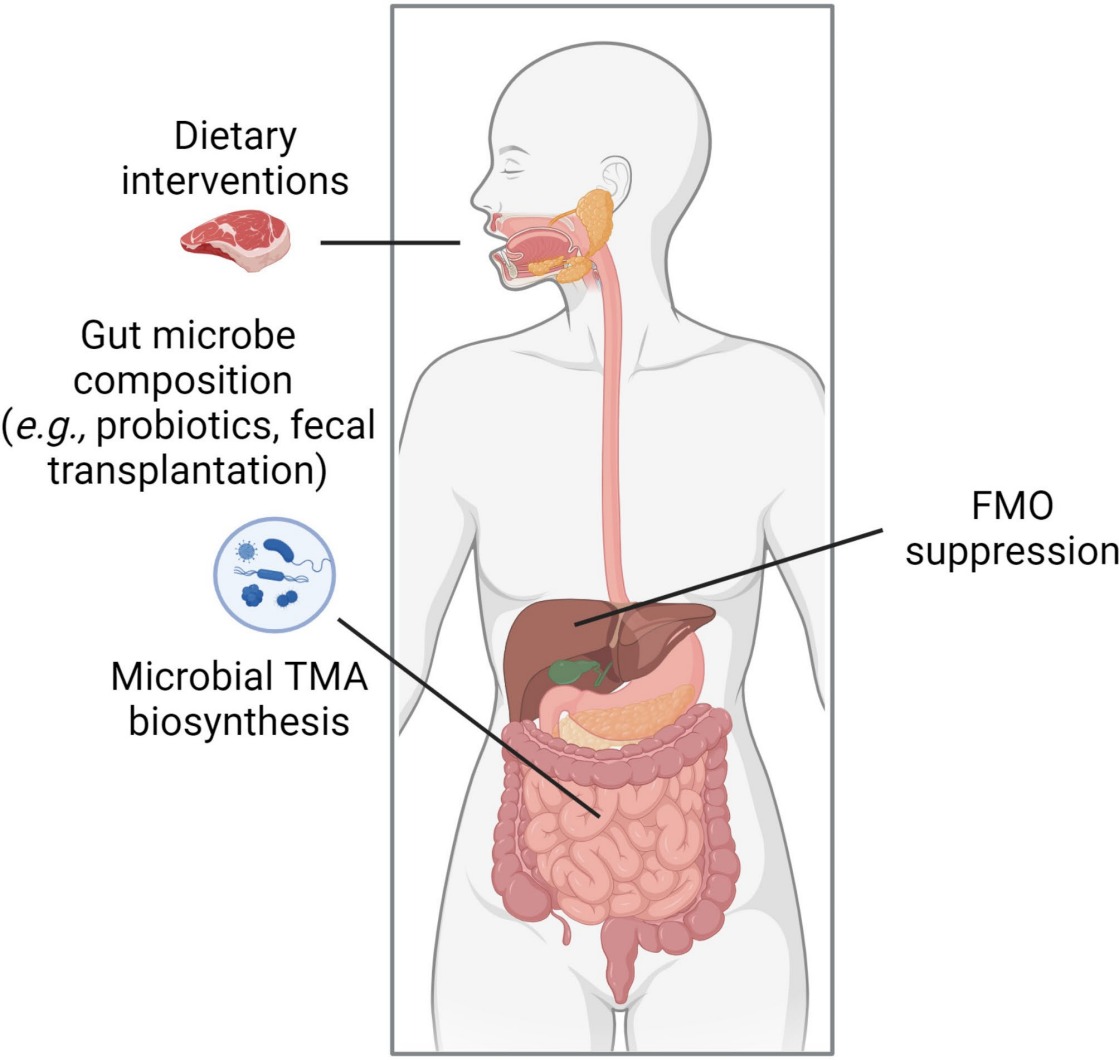
Therapeutic strategies involving TMAO include dietary interventions (e.g., reducing choline, L-carnitine, or betaine-containing compounds, dietary supplements, lifestyle modification), probiotics that alter TMA and TMAO levels by changing the gut microbial community, 3,3-dimethyl-1-butanol (DMB) or other CutC/D inhibitors that suppress microbial production of TMA, and inhibitors of FMO3

Inherent to the evidence supporting a direct causal link between TMAO and fibrosis in multiple organ systems is a presumption that modulation of TMAO could be a therapeutic strategy for preventing and treating fibrosis. As shown in Fig. 3, this could be achieved in several ways: dietary modification, modulation of the gut microbiome, or targeted approaches to selectively reduce TMA levels by reducing the conversion of dietary precursors into TMA.

Dietary interventions can be effective in lowering TMAO levels in humans. As an example, avoidance of red meat consumption was shown to reduce plasma TMAO levels within 4 weeks. (Wang et al. 2019) A red meat-rich diet increased TMAO levels through an increased supply of the TMA precursor carnitine, which elicited changes in the functional output of TMA by the gut microbial community and also lowered the renal excretion of TMAO. Reduced excretion of TMAO was reversed by a white meat and a non-meat diet. (Wang et al. 2019)

Another approach to reducing TMAO levels is to modulate the composition of the gut microbiota. Broad-spectrum antibiotics suppress production of TMA and TMAO, which, however, recover after the withdrawal of antibiotics. This strategy for lowering systemic TMAO levels has limited clinical potential since antibiotics broadly disrupt gut microbial ecology (Jernberg et al. 2007) and can lead to off-target effects on a myriad of host physiologic processes (Langdon et al. 2016) and antibiotic resistance. Probiotics (live microorganisms) or prebiotics (non-microbial substances that alter microbial community structure) also represent a potential strategy by altering the composition and the gut microbial community structure. Rats fed a high-fat diet and the probiotic *Enterococcus faecium* WEFA23 had reduced production of TMAO, (Huang et al. 2018) but the propensity for tissue fibrosis was not examined. Resveratrol, a natural polyphenol, was shown to decrease TMA production via gut microbiota remodeling in mice. On the other hand, treatment with resveratrol increased hepatic expression and activity of FMO3 and increased plasma TMAO levels following a single dose of TMA. (Chen et al. 2016a, b) Linaclotide, a GC-C agonist approved by the U.S. Food and Drug Administration for the treatment of constipation in irritable bowel syndrome, decreased the plasma levels of TMAO by modifying gut microbiota and reduced renal and cardiac fibrosis in choline-fed mice. (Nanto-Hara et al. 2020) Finally, transplantation of gut microbiota to regulate the proportions of specific high TMA-producing taxa is effective at reducing TMAO in mice. (Gregory et al. 2015)

Interfering with the ability of the host to convert microbial-derived TMA into TMAO would also lead to a decrease in circulating TMAO levels. Indeed, suppression of FMO3 has been shown to lower circulating TMAO levels and reduce diet-enhanced atherosclerosis in animal models. (Zhang et al. 2017; Miao et al. 2015) However, suppression of FMO3 has likely little clinical potential as inhibition of this enzyme has been shown to cause hepatic inflammation. (Warrier et al. 2015) Humans with inherited deficiency in FMO3 experience trimethylaminuria that is caused by the accumulation of TMA, resulting in a strong body odor. (Cashman et al. 2003)

Targeting TMA lyase activity in gut microbiota is another exciting strategy that appears to have potential as a clinical tool. Unlike antibiotics, small molecule inhibitors of TMA lyase like DMB and IMC are non-lethal to microbiota and likely exert less selective pressure for the development of resistance. DMB, a structural analogue of choline, was shown to inhibit microbial TMA lyases and reduce the level of TMAO in mice fed a high choline or carnitine diet. Treatment with DMB promoted reductions in the proportions of some microbial taxa that are associated with plasma TMA and TMAO levels. (Tang et al. 2015a, b) As discussed in previous sections, IMC has been shown to markedly suppress the production of TMA and lower plasma TMAO levels in animal models, (Gupta et al. 2020; Organ et al. 2016; Yang et al. 2019a, b; Roberts et al. 2018) suggesting that inhibition of TMA lyase activity can have a meaningful impact on host pathology. Taken together, these studies indicate strategies that inhibit TMA lyase activity and therefore TMA generation may serve as a therapeutic approach for the treatment of fibrotic diseases.

## Conclusions

Gut microbiota generate a plethora of biologically active metabolites that can exert a powerful direct or indirect influence on many aspects of host homeostasis and pathophysiology. Accumulating evidence support a strong link between elevated circulating TMAO levels and fibrosis in the heart, kidney, liver, and skin in diverse diseases. Thus, the nutrient-microbe-host meta-organismal framework linking diet and microbial metabolism to fibrosis represents a targetable pathway for therapeutics, and it will be interesting to see if selectively inhibiting microbial TMA lyase translates to a decreased propensity of fibrosis. The recent discovery of the first selective inhibitors of microbial TMA production (Tang et al. 2015a, b; Roberts et al. 2018) now opens the door for limiting TMAO exposure by “non-lethal drugging” of the gut microbiome (Brown and Hazen 2017) to reduce TMAO levels and prevent, slow, or reverse fibrosis.

### Electronic supplementary material

Below is the link to the electronic supplementary material.

## Data Availability

Not applicable.
